# Effect of a randomised exclusive breastfeeding counselling intervention nested into the MINIMat prenatal nutrition trial in Bangladesh

**DOI:** 10.1111/apa.13601

**Published:** 2016-10-28

**Authors:** Ashraful Islam Khan, Iqbal Kabir, Hanna Eneroth, Shams El Arifeen, Eva‐Charlotte Ekström, Edward A. Frongillo, Lars Åke Persson

**Affiliations:** ^1^ International Centre for Diarrhoeal Disease Research, Bangladesh (icddr,b)DhakaBangladesh; ^2^ International Maternal and Child HealthDepartment of Women's and Children's HealthUppsala UniversityUppsalaSweden; ^3^ Department of Health Promotion, Education and BehaviorArnold School of Public HealthUniversity of South CarolinaColumbiaSCUSA

**Keywords:** Counselling, Exclusive breastfeeding, Food and micronutrient supplementation, Pregnancy

## Abstract

**Aim:**

It is unknown whether maternal malnutrition reduces the effect of counselling on exclusive breastfeeding. This study evaluated the effect of breastfeeding counselling on the duration of exclusive breastfeeding, and whether the timing of prenatal food and different micronutrient supplements further prolonged this duration.

**Methods:**

Pregnant women in Matlab, Bangladesh, were randomised to receive daily food supplements of 600 kcal at nine weeks of gestation or at the standard 20 weeks. They also were allocated to either 30 mg of iron and 400 *μ*g folic acid, or the standard programme 60 mg of iron and folic acid or multiple micronutrients. At 30 weeks of gestation, 3188 women were randomised to receive either eight breastfeeding counselling sessions or the usual health messages.

**Results:**

The median duration of exclusive breastfeeding was 135 days in the counselling group and 75 days in the usual health message group (p < 0.001). Prenatal supplements did not modify the effects of counselling. Women in the usual health message group who were randomised to multiple micronutrients exclusively breastfed for 12 days longer than mothers receiving the standard iron–folate combination (p = 0.003).

**Conclusion:**

Breastfeeding counselling increased the duration of exclusive breastfeeding by 60 days. This duration was not influenced by the supplements.

**Key notes**

- Earlier studies of breastfeeding counselling have shown positive effects on duration of exclusive breastfeeding, but it is not known whether prenatal nutrition interventions increase this effect
- Eight counselling sessions increased exclusive breastfeeding by 60 days, but timing of prenatal food supplementation and different micronutrient alternatives did not increase the duration
- Among women who did not receive the breastfeeding counselling, prenatal multiple micronutrient supplementation was associated with 12 days longer exclusive breastfeeding that may be of small public health importance

## Introduction

Exclusive breastfeeding for the first six months of life, and continued breastfeeding together with adequate and safe complementary feeding for two years, is critical for physical well‐being, cognitive development and child survival. Exclusive breastfeeding until six months of age could reportedly prevent more than 800 000 child deaths worldwide every year 1.

In Bangladesh, around 90% of mothers are still breastfeeding their babies at one year of age and 88% are still breastfeeding at around two years 2. Despite breastfeeding promotional activities over the last two decades, the prevalence of exclusive breastfeeding at four to five months of age has remained relatively small, at 32% 2. Rapid urbanisation and female employment could partly explain the introduction of other foods before the age of six months. Many women in rural areas, and those who are not employed, introduce milk or semisolid foods to children at less than three months of age. Earlier studies reported that a high proportion of the mothers felt that they did not have enough breastmilk 3. Peers and other family members, such as mothers‐in‐law or elderly family members, could also influence perceptions of insufficient breastmilk.

Studies in Bangladesh have documented a significant increase in exclusive breastfeeding as a result of community‐based peer counselling 4 or breastfeeding counselling in hospitals 5. A systematic review and meta‐analysis confirmed that peer counselling enhanced the duration of exclusive breastfeeding, although the contextual influence of a formula‐feeding tradition might have decreased the effect 6. A wide range of counselling and breastfeeding promotion activities have been shown to increase the duration of exclusive breastfeeding 7. Continuous, repeated interactive sessions with counsellors in the health system were reported to be effective by a systematic review 8. Despite this, our knowledge is limited regarding the optimal timing, dose and mode of counselling that could be scaled‐up as an integrated part of the continuum of care provided by the health system to mothers and their children 8.

Furthermore, it has been questioned whether underweight mothers are less able to breastfeed their infants successfully 9. A review of multiple micronutrient supplements provided to breastfeeding women was inconclusive regarding the effects on breastfeeding performance due to the lack of good‐quality studies 10. To the best of our knowledge, there have been no previous studies that have evaluated the effect of prenatal food and multiple micronutrient supplementations on later breastfeeding performance.

The Maternal and Infant Nutrition Interventions (MINIMat) was a randomised trial in Matlab in rural Bangladesh (International Standard Randomised Control Trial Number: ISRCTN16581394). It was designed to examine the effect of combined food and micronutrient interventions on haemoglobin, birthweight and infant survival 11. An exclusive breastfeeding counselling intervention was nested into the trial and was provided to consenting mothers at around week 30 of pregnancy.

We postulated that in a population where being underweight was common, the early timing of prenatal food supplements combined with multiple micronutrients would enhance the effect of exclusive breastfeeding counselling.

The aim was to assess the effect of eight breastfeeding counselling sessions from late pregnancy to early infancy on the prevalence and duration of exclusive breastfeeding and to evaluate whether this effect was further increased by an early invitation to prenatal food supplements versus the usual timing and supplements with multiple micronutrients instead of the standard iron and folate combination.

## Methods

The MINIMat trial was conducted in Matlab, a rural subdistrict 57 km south‐east of Dhaka, the capital of Bangladesh. The International Centre for Diarrhoeal Diseases Research, Bangladesh (icddr,b), maintains the Health and Demographic Surveillance System in Matlab that monitors demographic and selected health information in the area. A total of 4436 pregnant women were enrolled from November 2001 to October 2003 and were randomised to the different nutritional interventions. The infants born to these women have been followed since birth. Written, informed consent was obtained from all of the participating mothers, and the study was approved by the Research and Ethical Review Committees of icddr,b.

### Study design

The MINIMat trial had a factorial design with two food supplement alternatives and three micronutrient supplements. At around week 30 of pregnancy, consenting women were allocated to breastfeeding counselling or received the usual antenatal healthcare messages. This study focuses on the effect of these interventions on the prevalence and duration of exclusive breastfeeding.

### Randomisation

In the MINIMat trial, all pregnant women were individually randomised to one of the food supplement groups and to one of the three micronutrient interventions, as previously described 11. The following eligibility criteria had to be met for enrolment: viable foetus, gestational age of less than 14 weeks by ultrasound examination, no severe illness and written consent for participation. At around nine weeks of pregnancy, women were randomly invited to receive food supplements at around nine weeks of gestation or to the standard programme where they could start the supplements when they chose, usually at around 20 weeks of gestation. Food supplements of 600 kcal per day was made available through community nutrition centres six days per week and continued up to the end of pregnancy. At 13 weeks of gestation, women started to receive the randomly allocated micronutrient supplementations that continued for their entire pregnancy: (i) 30 mg Fe fumarate and 400 *μ*g folate, (ii) the standard 60 mg Fe fumarate and 400 *μ*g folate or (iii) multiple micronutrients with 15 micronutrients including 30 mg Fe fumarate and 400 *μ*g folate. At around 30 weeks of gestation, simple random sampling was used to allocate consenting pregnant women to receive either counselling on exclusive breastfeeding or to receive the usual health message delivered by the regular icddr,b health staff during the antenatal clinic visits. The micronutrient supplementation was double‐blinded, while the food supplementation and the later breastfeeding counselling were randomly allocated but not blinded. An independent statistician performed all the randomisation procedures using a computer program. Randomisation lists were generated that included participant identity codes of the Health and Demographic Surveillance System and codes for the allocated interventions. Field staff, supervised by study supervisors, carried out the practical allocation to the different interventions. There were no deviations from the randomly decided allocations. Randomisation codes were safely kept at the administrative office of icddr,b and were not accessed until after performing the intent‐to‐treat analyses of the primary outcomes.

### Exclusive breastfeeding intervention

Nine women were recruited and trained as breastfeeding counsellors. Five were recruited at the start of the study, and another four joined after six months. They all lived in the local community, the Matlab study area, were married with at least one child, had breastfeeding experience and had a bachelor's degree equivalent to 14 years of schooling. One of the authors (IK) and one senior breastfeeding counsellor trained the other counsellors using a 40‐hour World Health Organization (WHO) and United Nations Children's Fund Breastfeeding Counselling Training module, which had been translated into the local Bangla language and adjusted to the local context. This training module had been successfully used in previous studies 4. The training was provided in daily four‐hour sessions over ten days. Counselling skills were taught using demonstrations and role‐play and included listening to mothers, learning about their difficulties, assessing the position and attachment of babies during breastfeeding, building mothers’ confidence, giving support and providing relevant information and practical help when required. The training also included practical sessions with pregnant women and mothers with infants. Two senior breastfeeding counsellors with at least six years of experience in counselling and one of the authors (IK) supervised the fieldwork and provided practical help at fortnightly sessions or via cell phones when required.

All women received basic healthcare messages, as is the usual practice in this region of Bangladesh. The women allocated to breastfeeding counselling received eight sessions: two sessions during the last trimester of pregnancy, one session in the seven days after delivery and five sessions at monthly intervals up to six months after childbirth. Counsellors were free to make additional contact if required. Counselling was given individually at home, but key family members were also included. The duration of each counselling visit was typically 20–40 minutes, depending upon the mother's lactation stage and her individual needs. Usually, the first two to three visits were longer, to build the mothers’ confidence, and the later counselling visits were shorter as they aimed to just give support to mothers and reinforce the health messages.

The women in the usual health message group received the messages on breastfeeding practices that were provided by the health staff of icddr,b when pregnant mothers visited the subcentres for their regular antenatal visits. These messages included the benefits of colostrum, advice on exclusive breastfeeding for four to six months and the recommended starting of complementary feeding from four to six months along with continuing breastfeeding for two years.

### Outcome data

Trained female interviewers collected data on infant feeding status every month up to 12 months of age. Data collection interviews were carried out one to three days before the counselling visits. A precoded semistructured feeding chart was developed, and feeding during the last 24 hours was recorded. Furthermore, the mother was asked whether the baby had received anything else besides breastmilk during the last month. If the mother had given the infant any other food or liquid than breastmilk for two consecutive days or more during the last month, this information was considered when classifying the feeding status and coded as partially breastfed.

### Feeding classification

The WHO definitions were used. To be classified as exclusive breastfeeding during the last month, only breastmilk could be given, not even water. Oral rehydration solution, liquid medicines and vitamins were allowed within the definition of exclusive breastfeeding.

### Other data

Maternal nutritional status and information regarding place and mode of delivery were extracted from the MINIMat databases 11. The socio‐economic characteristics of the households were determined using a continuous household asset score previously generated for this population, including data on land ownership, the construction materials used for house walls and the ownership of household assets. Data on the presence of such characteristics were analysed by principal component analysis, and a score was generated for each household, according to the principles that have been used by the World Bank and several other organisations 12.

### Data quality and management

An additional team cross‐checked one‐tenth of the interviewers’ scheduled visits to ensure the quality of the collected data. The questionnaires were checked daily, and, if the information was incomplete or not clear, a supervisor returned to the home the next day to complete and clarify the information. Data were stored in relational databases and further checked for any inconsistencies that were corrected in comprehensive data cleaning procedures.

### Statistical methods

The chi‐square test was used to compare the proportions receiving colostrum and the effect of the randomised breastfeeding intervention, namely the breastfeeding counselling versus the usual health message, on exclusive breastfeeding prevalence at four and six months. The total duration of exclusive breastfeeding was assessed in an intent‐to‐treat analysis, by including every participant in the analysis according to their initial randomisation. The analyses of the duration of exclusive breastfeeding were performed by analysis of variance (ANOVA). Interactions between prenatal food and micronutrient groups and the breastfeeding counselling intervention were analysed using product terms in ANOVA. First, a three‐way interaction was examined as follows: exclusive breastfeeding counselling × food supplementation × micronutrient supplements. If this was not statistically significant, two‐way interactions were examined as follows: exclusive breastfeeding counselling × food supplementation and exclusive breastfeeding counselling × micronutrient supplements. The significance level for interactions was set at p = 0.10.

### Sample size

The sample size for the MINIMat trial had been calculated for one of the primary outcomes, that is the effect of food and micronutrient supplements on birthweight. The sample size available for this study provided a power of 0.90 (type II error) and a probability of 0.05 (type I error) for a 6% difference in the prevalence of exclusive breastfeeding for the counselling group compared to the usual health message group. Such a difference was much smaller than those observed in previous studies. The sample size available for this study also provided a power of 0.88 (type II error) and a probability of 0.05 (type I error) for a 10% difference in the prevalence of exclusive breastfeeding for simple effect comparisons between any two of the six groups defined by the combination of the two counselling groups and the three micronutrient groups.

## Results

**Figure 1:**
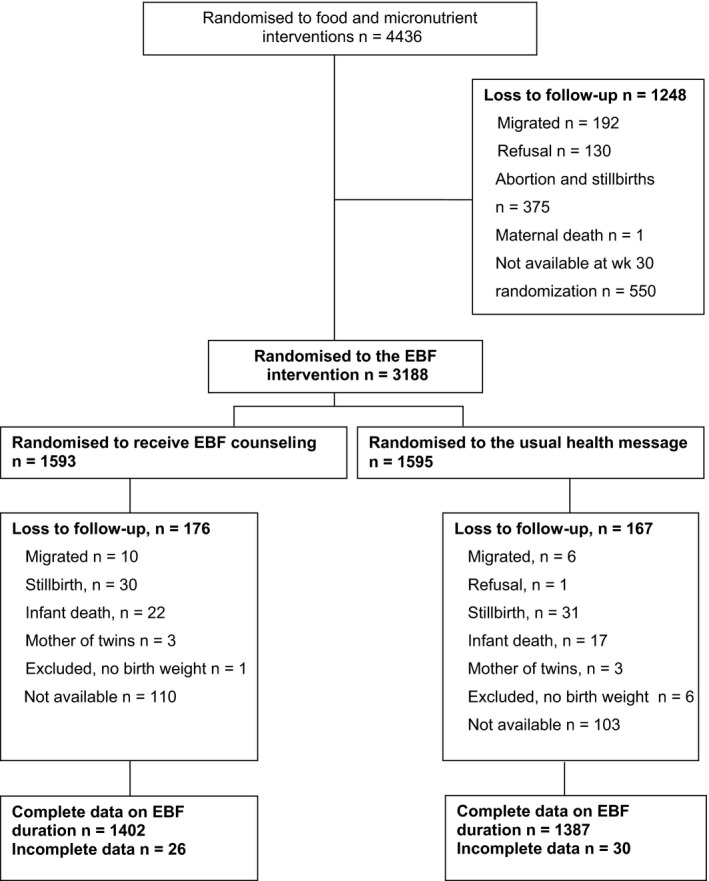
Study flow chart. The distribution of pregnant women and their children in the exclusive breastfeeding (EBF) counselling groups and the numbers lost to follow‐up during the Maternal and Infant Nutrition Interventions in Matlab (MINIMat) trial.

A total of 3188 mothers were randomised either to receive exclusive breastfeeding counselling from the counsellors or to receive the usual health messages from the Matlab regular health staff. The latter group served as the control. After omissions, 1387 mother–infant pairs in the counselling group and 1402 mother–infant pairs in the usual health message group completed six months of follow‐up. The distribution of pregnant women and their children in the exclusive breastfeeding counselling groups and the numbers lost to follow‐up are shown in Figure 1. The main reason for the women's lack of availability at the time of randomisation, at week 30, was the custom for pregnant women to visit their parents’ home at this time.

Mothers in the randomisation groups were similar at baseline (Table 1). The average age of the mothers was 26 years, their mean body weight was 45 kg, and their average height was 150 cm. Socio‐economic variables were similar across groups. The mean birthweights were 2690 and 2697 g and the birth lengths were 47.7 and 47.6 cm in the breastfeeding counselling and usual health message groups, respectively. About 57% and 56% had been delivered at home. The mean and standard deviation (SD) gestational age at birth was 38.7 ± 1.8 weeks and 38.7 ± 1.7 weeks in the breastfeeding counselling and usual health message groups, respectively.

**Table 1:** Baseline characteristics of mothers and infants at birth in the two intervention groups

Characteristic	Breastfeeding counselling group (n = 1387)	Usual health message group (n = 1402)
*Maternal*		
Age (years)	25.8 ± 6.0	25.7 ± 5.8
Education (mean number of years completed in formal education)	7.1 ± 2.9	7.2 ± 2.9
Asset score	−0.10 ± 2.30	0.06 ± 2.33
Asset score (n/n % in lowest quintile)	291/1417 (20.5)	278/1428 (19.5)
Asset score (n/n % in highest quintile)	260/1417 (18.3)	303/1428 (21.2)
Height (cm)	149.7 ± 5.3	149.8 ± 5.4
Weight at 8 weeks of gestation (kg)	45.1 ± 6.8	45.4 ± 6.9
BMI (at 8 weeks of gestation)	20.1 ± 2.7	20.2 ± 2.7
Place of delivery (n/n % at home)	808/1417 (57.0)	795/1428 (55.7)
Type of delivery (n/n % Caesarean section)	63/1417 (4.4)	59/1428 (4.1)
*Infant*		
Sex (n/n % female)	684/1417 (48.3)	699/1428 (48.9)
Birthweight (g)	2690 ± 415	2697 ± 409
Birth length (cm)	47.7 ± 2.2	47.7 ± 2.1

Data are mean (SD) unless indicated to be n/n (%).

In the breastfeeding counselling group, 95.5% of the babies had received colostrum as the first food, compared with 85.8% in the usual health message group (p < 0.001). By 72 hours, almost all babies in both groups had been breastfed. The median duration of exclusive breastfeeding was longer in the breastfeeding counselling group (135 days, 95% CI: 131–139) than in usual health message group (75 days, 95% CI: 68–82) – a difference of 60 days (p < 0.001). At four months, 69.0% (95% CI: 66.1–71.9) of the breastfeeding counselling group were being exclusive breastfed, as were 46.6% (95% CI: 42.8–50.4) in the usual health message group, while the corresponding figures at six months were 15.3% (95% CI: 10.4–20.1) and 6.4% (95% CI: 1.3–11.5), respectively.

There was no statistically significant three‐way interaction between breastfeeding counselling, food supplements and micronutrient supplements. The timing of the food supplements did not influence the effect by counselling on the duration of exclusive breastfeeding, with a p for interaction of 0.115 (Table 2).

**Table 2:** Analysis of breastfeeding counselling intervention with food and micronutrient supplementations on duration of exclusive breastfeeding

Interventions	n	Mean duration	95% CI	p value for interaction
Analysis of breastfeeding counselling × food supplementation				0.115
BFC + Early food	699	108.8	104.3–113.3	
BFC+ Usual food	688	113.7	109.2–118.1	
UHM+ Early food	708	77.3	72.8–81.8	
UHM+ Usual food	694	75.0	70.5–79.5	
Analysis of breastfeeding counselling × micronutrient supplementation				0.064
BFC+ Fe30	451	111.4	105.8–116.9	
BFC+ MMS	447	111.2	105.6–116.8	
BFC+ Fe60	489	111.1	105.8–116.4	
UHM+ Fe30	466	73.3	68.0–78.6	
UHM+ MMS	467	83.7	78.1–89.2	
UHM+ Fe60	469	71.6	66.0–77.1	

Interventions: BFC = breastfeeding counselling; UHM = usual health message; Early food = early invitation to food supplementation (around week 9), Usual food = usual timing of start of food supplementation (around week 20), Fe30 = 30 mg Fe and folic acid, Fe60 = 60 mg Fe and folic acid, MMS = multiple micronutrients.

Multiple micronutrient supplements did not further increase the effect of counselling on the duration of exclusive breastfeeding (Table 2). In the group of mothers who did not receive counselling, multiple micronutrient supplements were associated with longer exclusive breastfeeding of 12 days (p = 0.003).

## Discussion

We have shown that one‐to‐one breastfeeding counselling with eight sessions from pregnancy to six months after delivery with trained local counsellors resulted in higher prevalence and longer duration of two months of exclusive breastfeeding. The comparison group received the usual health messages that also included some breastfeeding promotion.

Data were longitudinally collected by interview at home every month, reducing the risk of recall bias. We also conducted a validation study using the deuterium dilution technique of mothers’ reported feeding behaviour and found a strong association between the questionnaire‐based information and the validation data, with positive predicted value of 0.84 13.

As shown in reviews of breastfeeding counselling interventions, continued counselling, support over time and contextual factors influence the magnitude of effects 6, 7, 8. In a previous peer‐counselling trial in urban Dhaka, the dose of the intervention, namely the number of counselling sessions, was almost double the number in the present study, and the size of the effect was larger 4. The present study showed that a lower number of contacts, close to the number of contacts a pregnant and delivering woman usually has with the regular maternal and infant health services, also resulted in a substantial increase in the duration of exclusive breastfeeding. The optimal number of counselling sessions across late pregnancy and early infancy is not known 6. Importantly, the training provided in this study emphasised the interactive character of the sessions 8 and the continuity of contact between the counsellor and the mother.

The total duration of exclusive breastfeeding in this trial may also have been influenced by the previous WHO guideline of exclusive breastfeeding four to six months that was still Bangladesh government policy at the time of the study.

Previous studies have asked whether marginally nourished women have the needed lactational capacity to breastfeed. A study in Bangladesh found evidence that the volume and quality of breastmilk in marginally nourished women may still be sufficient to ensure the healthy growth of their infants 9. A Cochrane review departed from the fact that many lactating women in low‐income settings suffer from multiple micronutrient deficiencies that might impair lactation performance. The authors found no trials, and only a few other studies, that addressed the possible impact of multiple micronutrients on breastfeeding outcomes and the result was nonconclusive 10. In this study, we hypothesised that *prenatal* multiple micronutrient supplementation versus iron–folate along with breastfeeding counselling would further increase the duration and rates of exclusive breastfeeding and providing prenatal food supplements earlier would further influence the effect in a positive way. In the group randomised to receive breastfeeding counselling, neither the multiple micronutrients nor the timing of the prenatal food supplementation affected exclusive breastfeeding duration. In the control group that was randomised to receive the usual health message, the allocation of multiple micronutrients was associated with longer duration of 12 days of exclusive breastfeeding. Even if this is valid, the public health importance of such a small difference might be limited.

## Conclusion

To conclude, the relatively limited number of eight counselling sessions with trained counsellors from pregnancy to the first part of infancy increased exclusive breastfeeding by two months. This is a ‘dose’ of the intervention that could be part of regular antenatal and child health services in order to achieve better adherence to the recommended duration of exclusive breastfeeding 1. However, the timing of a prenatal food supplementation and different micronutrient alternatives did not increase the effect of counselling. Among women who did not receive breastfeeding counselling, multiple micronutrients were associated with slightly longer exclusive breastfeeding that most likely may be of a small public health importance.

## Conflicts of interest

The authors declare that they have no competing interests.

## Funding

The MINIMat research study was funded by icddr,b, the United Nations Children's Fund, the Swedish International Development Cooperation Agency, the UK Medical Research Council, the Swedish Research Council, the Department for International Development, the Japan Society for the Promotion of Science, the Child Health and Nutrition Research Initiative, the Uppsala University and United States Agency for International Development. The funders had no role in any aspect of the study design or resulting manuscript.
